# Self-Assembly of Optimally Packed Cylindrical Clusters inside Spherical Shells

**DOI:** 10.1021/acs.jpcb.2c04850

**Published:** 2022-09-01

**Authors:** Horacio Serna, Ariel G. Meyra, Eva G. Noya, Wojciech T. Góźdź

**Affiliations:** † Institute of Physical Chemistry Polish Academy of Sciences, Kasprzaka 44/52, 01-224 Warsaw, Poland; ‡ IFLYSIB (UNLP,CONICET), 59 No. 789, B1900BTE La Plata, Argentina; § Departamento de Ingeniería Mecánica, UTN-FRLP, Av. 60 esq. 124, 1900 La Plata, Argentina; ∥ Instituto de Química Física Rocasolano, CSIC, C/Serrano 119, 28006 Madrid, Spain

## Abstract

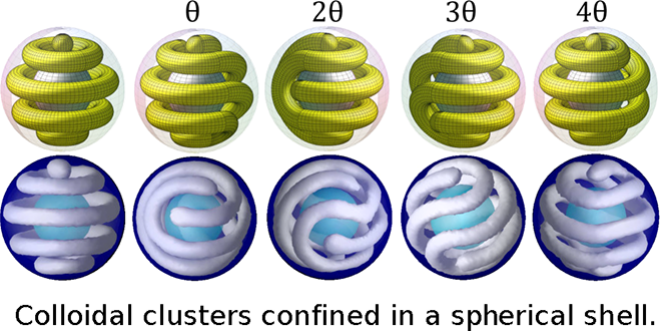

Systems with short-range attraction and long-range repulsion can form ordered microphases in bulk and under confinement. Using grand canonical Monte Carlo simulations, we study a colloidal system with competing interactions under confinement in narrow spherical shells at thermodynamic conditions at which the hexagonal phase of cylindrical clusters is stable in bulk. We observe spontaneous formation of different ordered structures. The results of the simulations are in a very good agreement with the predictions of a simple mathematical model based on the geometry and optimal packing of colloidal clusters. The results of the simulations and the explanation provided by a relatively simple geometric model may be helpful in manufacturing copolymer nanocapsules and may indicate possible ways of coiling DNA strands on spherical objects.

## Introduction

Systems with competing attractive and repulsive interactions are ubiquitous. Examples of these systems are block copolymers, mixtures of oil, surfactants and water, proteins, and colloidal suspension with depletants.^1,2^ When the ranges of interaction are properly tuned, systems with competing interactions can form a variety of ordered microphases such as cluster-crystals, hexagonal, bicontinuous, and lamellar phases. These ordered phases might be important in the development of new technological applications such as templating for nanomaterial synthesis,^3,4^ catalysis, drug delivery, and sensing.^5,6^ It has been demonstrated, by theory^1^ and simulations,^7,8^ that systems with competing interactions exhibit a universal phase behavior in bulk. Thus, the physical behavior observed in one such system can be discovered in other systems, and the results presented here are also relevant for a wide range of physical systems.

Recent studies have shown that by confining systems with competing interactions into pores with the proper geometry, new ordered microphases can be induced.^9−11^ The pore size must be carefully tuned to be commensurate with the periodicity of the ordered microphases to induce the formation of new ordered structures. In a previous study, we showed that the shape of the confining channels could strongly affect the structure of colloidal fluids with competing interactions.^9^

In this article, we study, by means of Monte Carlo simulations, the behavior of a colloidal fluid with short-range attraction and long-range repulsion (SALR) confined into narrow spherical shells. The geometry of the bulk phases in this system is incommensurate with the geometry of the spherical shell. We investigate how the cylindrical clusters that form the hexagonal phase are arranged in spherical shells of different sizes.

## Model and Simulation Details

**Figure 1:**
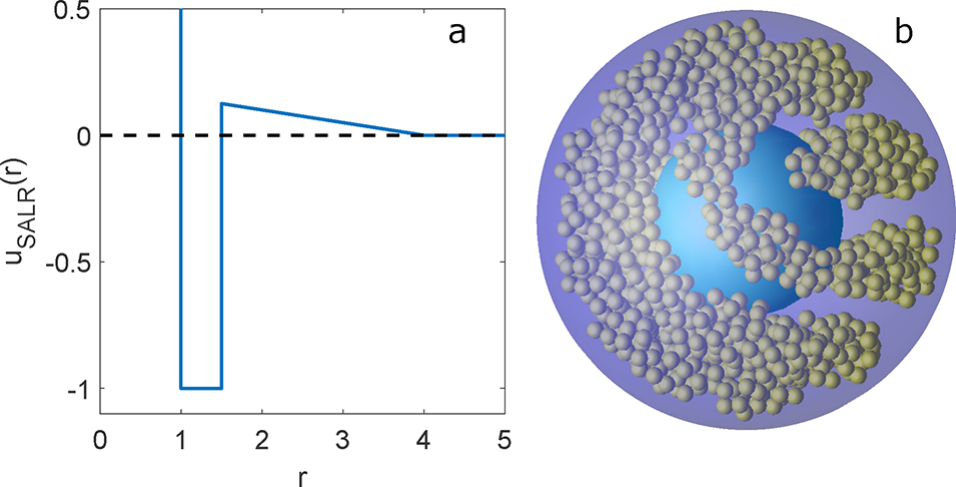
(a) Square-well-linear SALR potential used to model the interactions between colloidal particles. (b) Snapshot of the simulated system. The thermodynamic conditions are μ* = −2.170, T* = 0.35, *R*_inn_ = 6.0σ, and *R*_out_ = 11.0σ.

The colloidal system we model here is composed of mono-dispersed spherical particles that interact with each other via a SALR interaction potential. In particular, we consider the square-well-linear potential, consisting of a hard core, an attractive square-well, and a repulsive ramp. The pair potential is given by the following expression
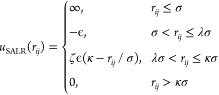
1Here, *r*_*ij*_ denotes the inter-distance between particles *i* and *j*, σ is the diameter of the colloidal particles, ϵ is the depth of the attractive well, λ is the attractive range, ζ denotes the repulsion strength, and κ is the repulsion range.^12,13^ The parameters are set to: ζ = 0.05, λ = 1.5, and κ = 4.0. The plot of the pair potential is presented in Figure 1a. For this set of parameters, the bulk phase diagram has already been calculated.^14^

The colloidal fluid is confined into spherical shells with hard walls. The system is finite and quasi two-dimensional since we focus on narrow shells. To construct such shells, we define an inner sphere of radius, *R*_inn_, and an outer sphere of radius, *R*_out_. Both spheres are concentric. The region between the two spheres defines the shell in which the particles are located at *S* = {*r*|*R*_inn_ ≤ *r* ≤ *R*_out_} where *r* is the radial coordinate. Based on the volume of the shell, the external potential is defined as
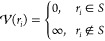
2Here, *r*_*i*_ denotes the radial coordinate of particle *i*. The total energy of the system is thus given by
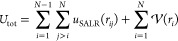
3*N* is the total number of colloidal particles.

The structure of the colloidal fluid confined in spherical shells is investigated by Monte Carlo simulations in the grand canonical ensemble (μ, *V*, *T*). All the values of the thermodynamics parameters (chemical potential, temperature, internal energy, density, and distance) are reported using σ and ϵ as units of distance and energy, respectively. The simulated systems contain between 1000 and 2200 particles. The length of the equilibration run depends on the system size. The production run, where averages are taken, consists of 2 × 10^10^ MC steps, from which 2 × 10^5^ independent configurations are taken for calculating the local density. A Monte Carlo step is defined as a trial move that may be a displacement, insertion, or deletion of a particle. We set the displacement attempt probability at 95% and the remaining 5% to the particle insertion or deletion attempts. Simulations are performed for the temperature T* = *k*_B_*T*/ϵ= 0.35 and for values of the chemical potential within the range −2.35 ≤ μ/ϵ = μ* ≤ −2.10. In these thermodynamic conditions, the hexagonal phase of cylindrical clusters is stable in bulk. We explore different system sizes in the range 6.0σ ≤ *R*_out_ ≤ 12.5σ. In a preliminary study, we found that the width of the spherical shell must be about 5σ to promote the formation of a single layer of cylindrical clusters. Thus, we set the shell width to *W* = *R*_out_ – *R*_inn_ = 5σ for all the studied cases. To calculate the number density of the system, ρ* = *N*σ^3^/*V*, we consider that the centers of the particles can be placed at the limits of the shell (i.e., at both *R*_inn_ and *R*_out_) and subtract and add σ/2 from the inner and outer radii, respectively, to account for the particle’s hard cores. Thus, the volume used for the calculation of the number density is, .

The structure of the colloidal fluid is identified by the visual inspection of iso-density surfaces built from the three-dimensional local density of the systems. These profiles are calculated by dividing the simulation box into small subvolumes [approximately (σ/2)^3^], measuring the particle density in each of these cells and averaging over 10,000 independent configurations. The iso-density surfaces are plotted for ρ_iso_ = 0.4 using OpenDX software. A snapshot of the configuration taken from the simulations is shown in Figure 1b.

## Results and Discussion

**Figure 2:**
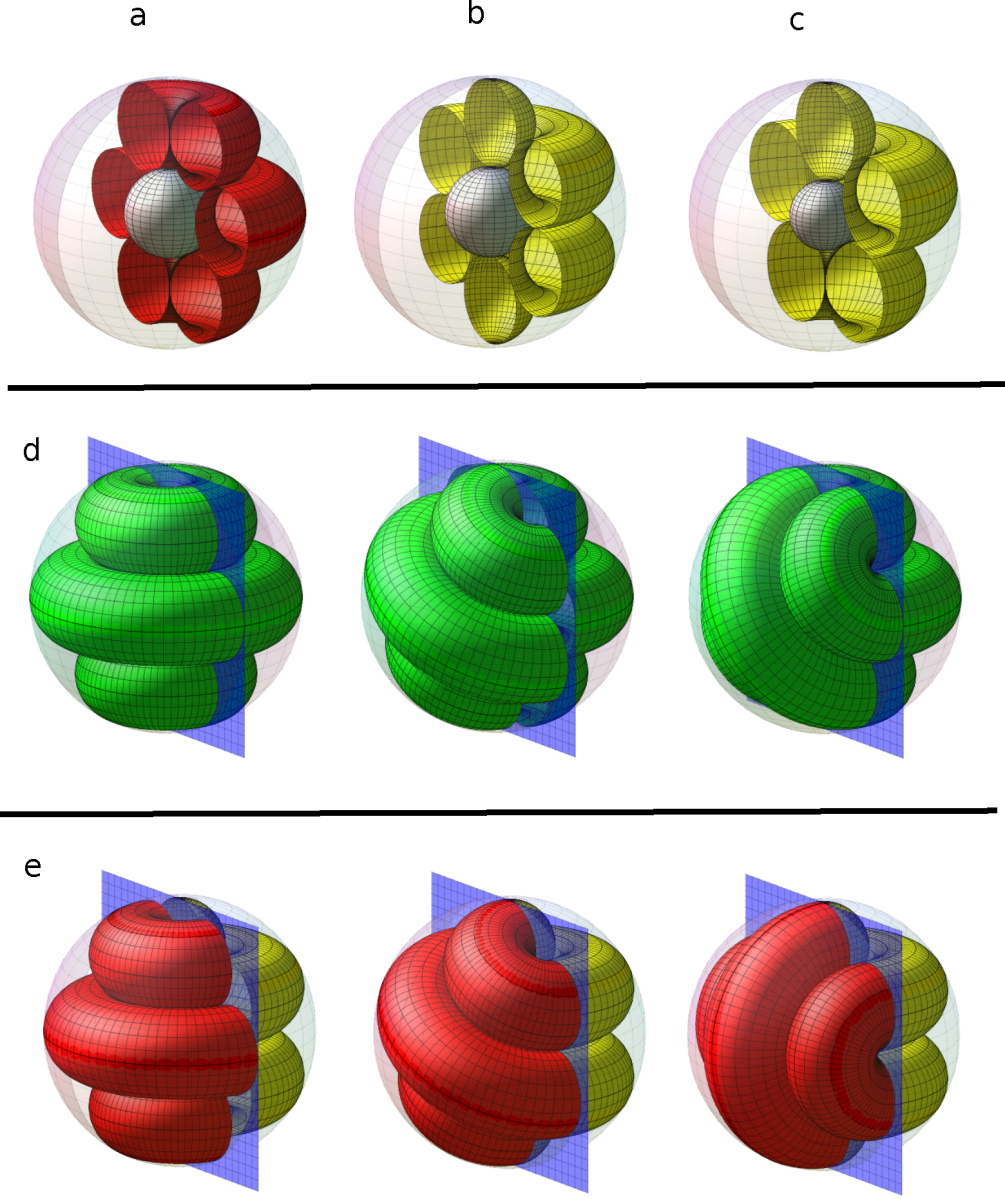
Examples of closed packing of tori and spheres in a shell of a fixed width. (a) Three tori, (b) two tori and two spheres, and (c) one sphere and two tori. The diameter of the tori and spheres is equal to the width of the shell. (d) Construction of new structures from the configuration shown in (a) (in red) by rotating one hemisphere by an angle θ/2 (rotation at the intermediate position) and θ (new structure formed after a complete rotation). (e) Construction of two hybrid structures made by joining the hemispheres shown in (a,b) (in red and in yellow) and by rotating one of these hemispheres by an angle θ/2 and 3θ/2, respectively.

We have noticed that many structures obtained in the simulations can be constructed considering a relatively simple mathematical model. Consider a shell composed of two concentric spheres with radii *R*_inn_ and *R*_out_, *R*_inn_ < *R*_out_, where *W* = *R*_out_ – *R*_in_ is the width of the shell. We want to fill the volume of the shell with spheres and tori of diameter *W*. For fixed width *W*, it is possible to calculate the values of the radii *R*_inn_ and *R*_out_ to obtain optimal packing of the shell with tori and spheres, as illustrated in Figure 2. There are three possible arrangements of tori and spheres inside the shell to obtain an optimal packing. The shell can be filled with: (a) only tori, (b) tori and two spheres, and (c) tori and one sphere. The number of tori and spheres depends on the values of *R*_inn_ and *R*_out_. It is interesting to note that the closed packed structures composed of *k* tori or *k* – 1 tori and two spheres are obtained for the same size of the shell as shown in Figure 2a,b. The structures composed of only tori and spheres can be used to generate many other closed packed configurations. To obtain new structures, the shell is cut through a plane containing the rotation axis, as shown in Figure 2. Next, one hemisphere is rotated around the axis passing through the center and perpendicular to the cutting plane, as shown in Figure 2d. For a specific set of the rotation angle, open ends of the tori forming two hemispheres can be smoothly connected. The rotation angle can be calculated by dividing 2π by the number of open toroidal ends and open small hemispheres. Each cut torus has two open ends. When the closed packed structure is built of *k* tori and *l* spheres (with *l* = 1 or 2), then the angle of rotation θ can be calculated according to the following formula θ = 2π/(2*k* + *l*). With the method we have described to obtain the derived structures, both right- and left-handed structures can be constructed. Although we are just presenting structures obtained in simulations with one handedness, we often observed the formation of both chiral structures.

The inner radius of the shell *R*_inn_ for a given number 2*k* + *l* of closed packed torii and spheres and the shell width *W* can be calculated from the following formula: .

Since the sizes of the systems built of *k* tori or *k* – 1 tori and two spheres are the same, it is possible to combine hemispheres of two different arrangements of tori and spheres to obtain new closed packed structures. In such a case, the angle of the first rotation is θ/2. We call such structures the hybrid ones (see Figure 2e). They are pictured in the figures presented in this article with two colors, red and yellow. A similar method has been already used to predict the possible solutions to the problem of finding the longest rope on the surface of a sphere.^15^

The cylindrical and spherical clusters obtained in the simulations of the SALR model are not rigid and, due to the long-range repulsion, these clusters repel each other. Loosely speaking, this soft repulsion plays a similar role to the hard core repulsion in the previously discussed mathematical model. In the case of the hexagonal phase made of cylindrical clusters, the distance between the centers of the neighboring clusters can be considered as equivalent to the size of the diameter of hard tori. Thus, one might expect that the structures calculated in the mathematical model may also be obtained in the simulations. It has to be noted that the local density of the colloidal particles in the middle distance between the clusters is close to zero. In the figures presented in this article, the configurations from the mathematical model are compared with the configurations from the simulations. We decrease the diameter of the tori and visualize the iso-density surface at ρ_iso_ = 0.4 for easier identification of the corresponding configurations. In the simulations, the radius of the clusters (spheres and tori) and the separation distance between them are determined by the interplay between attraction and repulsion. The separation distance between clusters must be close to that of the lattice constant of the hexagonal phase of cylindrical clusters in bulk, *l*_0_ ≈ 6σ, and the radius of the clusters to that of the equilibrium radius of the cylindrical clusters, *r*_0_ ≈ 1.5σ.^9,10^

### Simulations

For very small spherical shells (*R*_out_ of the order of *l*_0_), the colloidal particles on the opposite sides of the limiting inner sphere are within the repulsive range of the interparticle potential and the ordered structures that form instantaneously have short life times at T* = 0.35. Consequently, we found that the minimum limiting radius of the inner sphere that allows for the formation of ordered structures at this temperature is approximately *R*_inn_ = 1.5σ. Below this limit, colloidal particles located near the surface of the inner sphere in diametrically opposite positions start to experience some repulsion because the distance between them is lower than the range of the repulsive interactions (see eq 1 and Figure 1).

**Figure 3:**
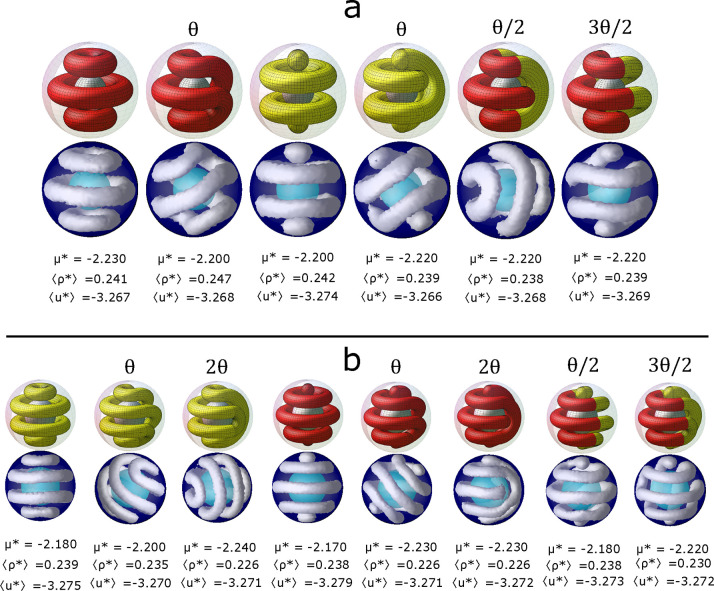
Ordered structures obtained from simulations by confining the colloidal fluid into a small spherical shell of (a) *R*_inn_ = 3.0σ and *R*_out_ = 8.0σ and (b) *R*_inn_ = 5σ and *R*_out_ = 10σ. The temperature is T* = 0.35. The chemical potential, (μ*), the average number density, (⟨ρ*⟩), and the average energy, (⟨u*⟩), are shown in the figure. The iso-density surfaces are presented in gray with ρ_iso_ = 0.4, and the outer and inner spheres are shown in blue and cyan, respectively. The corresponding structures pictured in red and yellow are obtained from the mathematical model. Hybrid structures are pictured in two colors. The angle of rotation θ is π/3 for (a) and π/4 for (b). The corresponding angles for each structure are shown in the figure.

We first discuss the results obtained for small spherical shells. In Figure 3a, we present all the possible structures for a shell with *R*_inn_ = 3.0σ and *R*_out_ = 8.0σ. We note that for the same chemical potential, it is possible to obtain different structures. We observe single and double helices, toroidal structures, structures formed by a combination of toroidal and spherical clusters, by a cylindrical cluster forming closed loops, by a cluster with two open ends, and by a combination of closed and open clusters. Note that all these structures can be derived from the previously discussed mathematical model. In a recent study of SALR disks confined to the surface of a sphere, structures very similar to the ones considered by us have been reported.^16^

In Figure 3b, we present a more complex example of the larger hybrid structure for the spherical shell of *R*_inn_ = 5σ and *R*_out_ = 10σ. The simulations predict the stability of the structure with four tori and the structure with two spheres at the poles and three tori for μ* = −2.180 and −2.170, respectively, with very similar densities and energies. All the possible structures derived from the mathematical model are obtained from the simulations within a small range of chemical potential. Hybrid structures composed of two different arrangements of closed packed tori and spheres can also be obtained by rotating the hemispheres by odd multiples of θ/2. The hybrid structures are pictured in two colors (red and yellow), representing hemispheres of two different structures.

**Figure 4:**
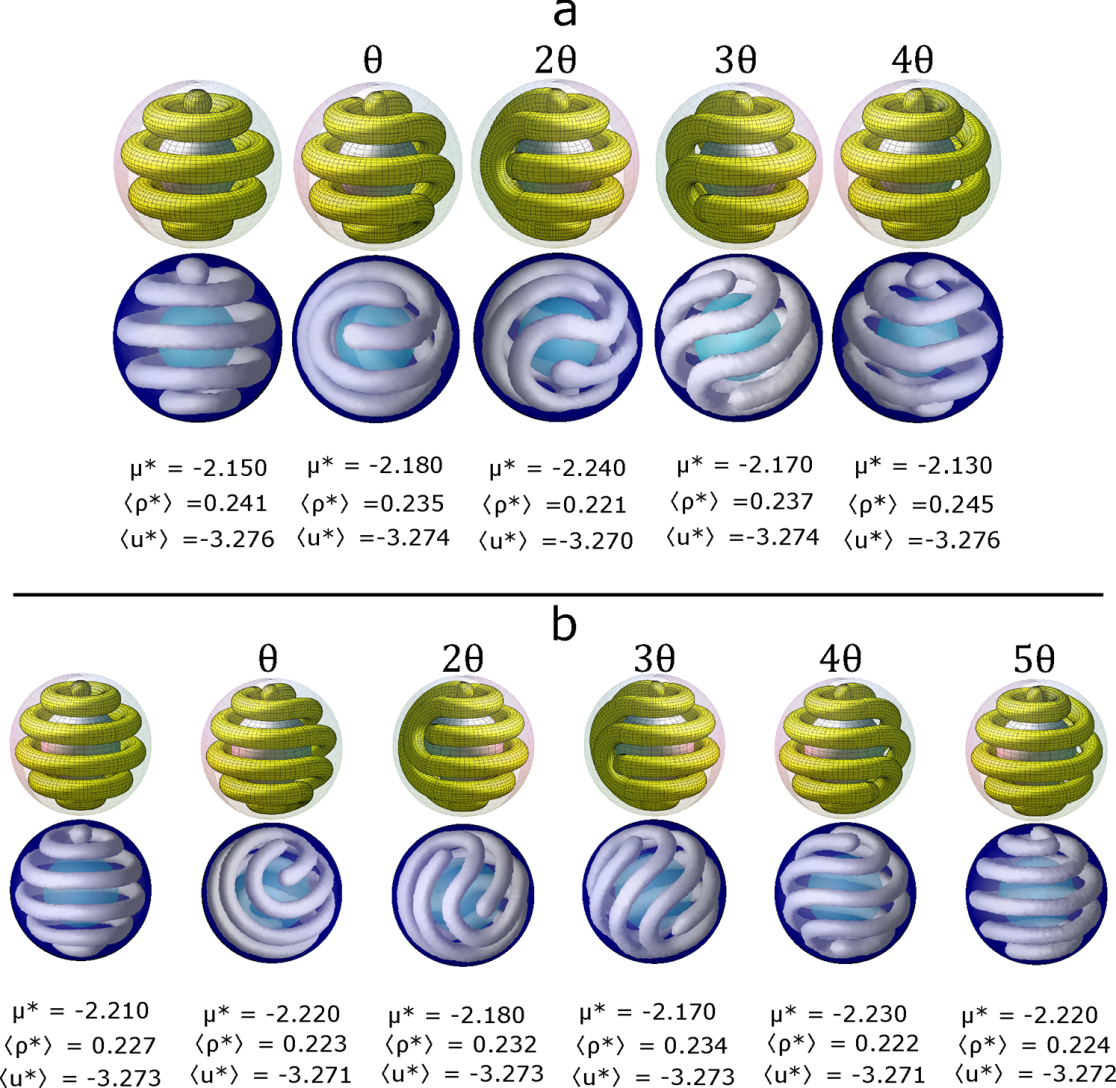
Structures obtained from simulations by confining the colloidal fluid into a spherical shell of (a) *R*_inn_ = 6σ and *R*_out_ = 11σ and (b) *R*_inn_ = 7.5σ and *R*_out_ = 12.5σ. The temperature is T* = 0.35 and the chemical potential, μ*, the average number density, (⟨ρ*⟩), and energy, (⟨u*⟩), are presented for each structure. The iso-density surfaces are presented in gray with ρ_iso_ = 0.40, and the outer and inner spheres are shown in blue and cyan, respectively. The corresponding structures pictured in yellow are obtained from the mathematical model. The first structure is successively rotated by the proper angle θ, 2π/9 for (a) and 2π/11 for (b), to form all the derived structures.

Figure 4a shows all the possible structures obtained for the spherical shell with *R*_inn_ = 6σ and *R*_out_ = 11σ. The generating structure is composed of one spherical cluster and four toroidal clusters. The derived structures are obtained by rotating one of the hemispheres of the generating structure by multiple integers of an angle, θ = 2π/9. Interestingly, we observe that the average energies of all the structures are quite similar. The number densities of the systems are also very similar, but they increase as the chemical potential increases. The derived structures corresponding to rotations of θ, 2θ, and 4θ are composed of only one coiled cylindrical cluster. The structure corresponding to a rotation of 3θ is composed of two clusters. The first one is an open cluster resembling a single-helix, and the second one is a closed cluster. In the θ structure, the two ends of the cylindrical cluster are placed at the same pole. This structure resembles a double helix that closes on itself at the opposite pole. In the 2θ structure, the ends of the cylindrical cluster are separated by one of its folds. Two folds separate the ends of the 3θ structure, but, as already mentioned, the structure is composed of one open cluster and one closed cluster. Finally, the 4θ structure has its ends placed at opposite poles separated by four folds. This structure resembles a single helix structure.

In Figure 4b, we show all the structures obtained for a shell of *R*_inn_ = 7.5σ and *R*_out_ = 12.5σ. The generating structure is composed of one spherical cluster and five toroidal clusters. Besides the fact that the average energies and densities are very similar for all the structures, we found that two different structures can be obtained at exactly the same thermodynamic conditions (see second and sixth structures in Figure 4b), suggesting that all the possible structures can exist for a similar range of the chemical potentials within the range of stability of the hexagonal phase of cylindrical clusters in bulk at T* = 0.35. Unlike the *R*_out_ = 11σ shell, all the derived structures are formed by only one open coiled cylindrical cluster. The derived structures follow the same pattern as in the previous case; the ends of the open cluster are placed first at the same pole for a rotation of θ, then the ends are separated by one fold for 2θ and so on.

It should be stressed that the structures obtained in the simulations for a given size of the shell are related by geometrical transformations. Therefore, it is natural to expect that the properties such as energy and density are comparable for the related structures. The transitions between the related structures are also possible if the energy barrier between the minima is not very large. In fact, in long simulations we have observed such transitions. However, when the structure is formed in a simulation, it is stable for a long time, and we are easily able to obtain statistically accurate results for energy, average, and local density. The structures are formed from random configurations. No special initial configuration is needed to facilitate the formation of the obtained structures.

## Summary and Conclusions

We have studied self-assembly of a colloidal system with competing interactions confined in narrow spherical shells of constant width *W* = 5σ, at thermodynamic conditions, μ, *V*, and *T*, at which the hexagonal phase of cylindrical clusters is stable in bulk. All the possible structures for a given shell size have very similar average densities and energies and are obtained in a narrow range of chemical potentials. We found that different structures can be obtained at the same thermodynamic conditions (see, e.g., Figure 4b, for rotations of θ and 5θ, and some structures presented in Figure 3). We also observed that the fluctuations of the number of particles often drive the transitions from one structure to another.

The existence of the structures observed in simulations can be explained based on a simple mathematical model where only the geometry of the system is taken into account. Interestingly, the structures reported in this paper are analogous to the solutions to the problem of the longest rope on the surface of a sphere. Based on the results of the simulations, we found that the system self-assembles into a new type of hybrid structures, composed of two hemispheres corresponding to different arrangements of tori and spheres (see Figure 3). We were able to construct such closed packed structures in the mathematical model as well (see Figure 2). Thus, we demonstrated how the simulations of a physical system can help find the solutions of some mathematical problems.

The structures described in this article could be realized experimentally with block-copolymers adsorbed on spherical particles. The length and composition of the co-polymer must be finely designed to obtain the appropriate ranges of interactions with respect to the size of the adsorbing particles. Similar structures have been reported in the experiments with thin block-copolymer films adsorbed on the surface of colloidal particles.^17^

Likewise, theoretical calculations have predicted that the stability of helical and toroidal structures in asymmetric diblock copolymers confined in small spherical cavities^18−20^ or core–shell particles with copolymers forming a shell around a core built form a homopolymer.^21^ In a fluid of SALR particles confined to the surface of a sphere, helical structures have also been obtained in the density functional theory calculations^16^ and simulations.^22^ In our study, we are using a SALR potential with a flat minimum and are modeling a quasi-two-dimensional system, whereas in refs (16) and (22), a SALR potential with a very sharp minimum is used, and the system is strictly two-dimensional. Since different SALR systems behave in a universal way, one would expect that the structures presented in this article can be observed in other such systems.

The ordered structures presented in this article are not exclusive to equilibrium systems. The same structures can be observed in Turing patterns obtained by solving an appropriate reaction-diffusion system on the surface of a sphere.^23−25^ We believe that the results presented in this paper might be important to the conceptual design of soft nanomaterials that involve systems with competing interactions and spherical geometries.
